# The Role of Ca^2+^ Signaling in Aging and Neurodegeneration: Insights from *Caenorhabditis elegans* Models

**DOI:** 10.3390/cells9010204

**Published:** 2020-01-14

**Authors:** Javier Alvarez, Pilar Alvarez-Illera, Paloma García-Casas, Rosalba I. Fonteriz, Mayte Montero

**Affiliations:** Institute of Biology and Molecular Genetics (IBGM), Department of Biochemistry and Molecular Biology and Physiology, Faculty of Medicine, University of Valladolid and CSIC, Ramón y Cajal 7, E-47005 Valladolid, Spain; pilar_alvill@hotmail.com (P.A.-I.); palomabio21@gmail.com (P.G.-C.); rfonteri@ibgm.uva.es (R.I.F.); mmontero@ibgm.uva.es (M.M.)

**Keywords:** Ca^2+^, *C. elegans*, neurodegeneration, Alzheimer, Parkinson, SERCA, IP_3_ receptor, ryanodine receptor

## Abstract

Ca^2+^ is a ubiquitous second messenger that plays an essential role in physiological processes such as muscle contraction, neuronal secretion, and cell proliferation or differentiation. There is ample evidence that the dysregulation of Ca^2+^ signaling is one of the key events in the development of neurodegenerative processes, an idea called the “calcium hypothesis” of neurodegeneration. *Caenorhabditis elegans* (*C. elegans*) is a very good model for the study of aging and neurodegeneration. In fact, many of the signaling pathways involved in longevity were first discovered in this nematode, and many models of neurodegenerative diseases have also been developed therein, either through mutations in the worm genome or by expressing human proteins involved in neurodegeneration (β-amyloid, α-synuclein, polyglutamine, or others) in defined worm tissues. The worm is completely transparent throughout its whole life, which makes it possible to carry out Ca^2+^ dynamics studies in vivo at any time, by expressing Ca^2+^ fluorescent probes in defined worm tissues, and even in specific organelles such as mitochondria. This review will summarize the evidence obtained using this model organism to understand the role of Ca^2+^ signaling in aging and neurodegeneration.

## 1. Introduction

Studying human diseases always requires the choice of the best possible model. Of course, humans would be the best subjects to study, but there are practical and ethical limitations that generally prevent them from being used as study subjects. The next obvious choice is other mammals, particularly mice, and they are excellent in many ways, particularly when mimicking or studying behavioral phenotypes. However, they also have important limitations, both in how well they mimic some diseases, and in the slow development of pathologies and the time required to obtain conclusions. This is specially the case for the neurodegenerative diseases that develop at an advanced age, requiring several months of study in mice. On the other hand, disease models in cell cultures offer the advantage of ease of handling and the possibility of full genetic and metabolic manipulation. However, it is much more difficult to obtain information from these studies which is able to be easily translated into the physiological response of an entire organism.

Models of invertebrate organisms such as the fly *Drosophila melanogaster* or the nematode *C. elegans* constitute a reasonable intermediate that has been widely used in recent years, as they combine the ease of study with the presence of a complete organism, albeit with the obvious limitation of the greater difficulty in mimicking human disease.

The nematode *C. elegans* was introduced as a model organism by Sydney Brenner in 1963 [1]. It has a three-day life cycle from egg to adult and a lifespan of only about 20 days, which facilitates studies of aging. It has two sexes, i.e., XX hermaphrodites and XO males; the hermaphrodites can reproduce either by self-fertilization or by mating with males. A hermaphrodite can lay about 300 eggs after self-fertilization, and more than 1000 after mating with males. It is therefore possible to easily obtain large populations of identical worms.

*C. elegans* was the first multicellular organism to have completed the genome sequence in 1998, and the complete lineage of its 959 somatic cells in the hermaphrodite (including 302 neurons) is also known [2]. It is also the first organism in which all the neuronal connections in both sexes have been mapped, providing complete nervous system connectomes [3]. As it is a completely transparent organism throughout its life, it is possible to visualize and monitor specific cell types by expressing targeted fluorescent proteins such as green fluorescent protein (GFP) derivatives or fluorescent Ca^2+^ sensors.

In spite of the large differences in the evolutionary scale, comparative proteomics indicates that 83% of the *C. elegans* proteome comprises human homologous genes [4]. Comparative genomic analysis also shows that nearly 53% of the human protein-coding genome has recognizable worm orthologues [5]. In addition, genetic manipulation is relatively easy. Thus, for those human genes having no orthologue in the worm, it is possible to generate “humanized” worms expressing the human gene, either wild-type or mutated, in defined tissues. Thanks to all these characteristics, *C. elegans* offers many advantages for studies of human diseases, and in particular, neurodegenerative diseases. The first worm model of Alzheimer’s disease was made in 1995 [6], and since then, worm models of most neurodegenerative diseases have been generated (see below). These models have been used to study the molecular alterations present in these pathologies, but also to perform high-throughput drug screening to identify chemical compounds with neuroprotective activity [7,8].

In this review, we will discuss in detail the most recent data obtained from these models on the role of Ca^2+^ signaling in neurodegeneration. Before that, we will first briefly summarize the main models of neurodegenerative diseases in *C. elegans* and the present knowledge on the role of Ca^2+^ signaling in these pathologies.

## 2. Models to Study Neurodegenerative Diseases in *C. elegans*

### 2.1. Alzheimer’s Disease

Alzheimer’s disease is the most common form of dementia, and is histologically characterized by the presence of deposits of amyloid plaques containing the β-amyloid (Aβ) peptide and neurofibrillary tangles containing hyperphosphorylated tau protein. The Aβ peptide is generated by the cleavage of the precursor amyloid precursor protein (APP) through the proteases known as β-secretase and γ-secretase. Mutations in APP or in the presenilin proteins (PS1 or PS2), which are components of the γ-secretase, produce familiar forms of the disease. Mutations in the tau gene, instead, produce a different neurodegenerative disease, called frontotemporal dementia.

*C. elegans* has an orthologous of the APP gene, called *apl-1*, but the APL-1 protein lacks the Aβ peptide. In addition, the worm genome does not contain β-secretase. Therefore, although *apl-1* mutants have been used to study the physiological role of the APP protein, *C. elegans* models of Aβ toxicity have always been made by expressing the human Aβ peptide in the worms. The first *C. elegans* model of a neurodegenerative disease was made by expressing the Aβ_3-42_ peptide in body-wall muscle [6,9]. The resulting transgenic worms suffered paralysis, and characteristic Aβ deposits were observed in the muscles. Several worm models with either muscle or neuronal Aβ_1-42_ or Aβ_3-42_ expression have since been made, and they have also been used to investigate the neuroprotective effect of several compounds [7,9,10,11,12,13,14].

*C. elegans* has three presenilin genes: *sel-12, hop-1* and *spe-4* [9,11,15]. *spe-4* is only expressed in the male germ line, while *hop-1* and *sel-12* are widely expressed and *sel-12* shows the higher sequence homology to human presenilins. In the same way as presenilins control APP processing, SEL-12 appears to regulate APL-1 cleavage, because *apl-1* overexpression produces 70% lethality, and this is rescued in *sel-12* mutants [16]. Mutations in *sel-12* result in mitochondrial metabolic defects that promote neurodegeneration in the worm as a result of deregulation of mitochondrial Ca^2+^ homeostasis [15,17,18] (see below Section 4.1.).

*C. elegans* has only one orthologue of tau known as *ptl-1*, which has a high degree of sequence homology with mammalian tau. There is evidence that *ptl-1*, as human tau, plays an important role in maintaining neuronal integrity [9,19]. Mutations in tau produce frontotemporal dementia, and worm models with transgenic expression of mutated human tau variants produce a progressive uncoordinated phenotype and neurodegeneration [9,19].

### 2.2. Parkinson’s Disease

Parkinson’s disease is the second most frequent neurodegenerative disease after Alzheimer’s disease. It is a disorder characterized by a progressive impairment in the control of body movements, as a result of the death of dopaminergic neurons in the *substantia nigra* of the brain. At the cellular level, the disease is characterized by the accumulation of the α-synuclein protein into neuronal inclusions called Lewy bodies. Although most cases of Parkinson’s disease are sporadic, 5–10% are familial. These cases have been associated with mutations in several genes, including α-synuclein, the vesicular trafficking protein VPS35, the multi-domain kinase LRRK2, and the mitochondrial stress response proteins PINK1, Parkin, and DJ-1. The *C. elegans* genome includes orthologues for most of these genes, except for α-synuclein [9,20,21,22,23].

α-synuclein is an aggregation-prone neuronal protein whose cellular function is not well known. As *C. elegans* has no orthologue of this protein, worm models have been generated by overexpression of wild-type or mutant forms of human α-synuclein in different tissues, i.e., either body-wall muscle, pan-neuronal, or only in the dopaminergic neurons. In most cases, overexpression leads to locomotion defects and the degeneration of dopaminergic neurons [9,20,21,22,23].

Research on Parkinson’s disease in *C. elegans* has also been made by studying the effects of mutation of the worm orthologues of PINK1 (*pink-1*), LRRK2 (*lrk-1*), PARKIN (*pdr-1*), and DJ-1 (*dnaj-1.1* and *dnaj-1.2*). Further, in some cases, the effect of overexpression of the pathogenic mutant form of the human protein has been studied. For example, overexpression of a mutant form of human LRRK2 also induces age-dependent neurodegeneration in the worms [9,20,21,22,23].

Toxin-induced models of Parkinson’s disease have also been developed by the administration to the worms of several neurotoxins, such as 1-methyl-4-phenyl- 1, 2, 3, 6-tetrahydropyridine (MPTP), 6-hydroxydopamine (6-OHDA), rotenone, paraquat, or manganese [20,21,23]. The availability of *C. elegans* strains with different tissues or selected neuronal populations specifically labelled with fluorescent proteins or Ca^2+^ sensors allows direct visualization of the changes in the cellular morphology or Ca^2+^ dynamics induced by the toxins.

### 2.3. Huntington’s Disease

Huntington’s disease is a familial progressive neurodegenerative disease caused by an autosomal dominant mutation in the huntingtin gene that produces a protein, named huntingtin, whose function remains unclear. Pathological forms of huntingtin have an expansion of the CAG triplet coding for glutamine in the N-terminal polyglutamine region of the protein, and full penetrance of the disease occurs above 40 glutamines in that region.

As *C. elegans* lacks any huntingtin homolog, models of this disease have been created by expressing N-terminal fragments of human huntingtin, with different numbers of glutamines in the polyglutamine region, in specific neuronal populations. The models show that also in *C. elegans*, 40 glutamine repeats seems to be the critical number to generate protein aggregation and pathology [7,9,14].

### 2.4. Amyotrophic Lateral Sclerosis

Amyotrophic lateral sclerosis is a neurodegenerative disorder characterized by progressive body paralysis due to damage of motor neurons in brain and spinal cord. About 10% of the cases are familial, and they have been associated with mutations in more than 20 different genes. The most frequent mutations are found in C9orf72 (10–15% of familial cases), the superoxide dismutase SOD1 enzyme (2%), the TDP-43 protein, which is a component of the ubiquitin-containing aggregates that appear in the motor neurons (0.9%), and the FUS protein (RNA-binding protein Fused in Sarcoma, 0.7%), although mutations in many other genes have been found to be associated with this disease. *C. elegans* has orthologues of all these four genes, and *C. elegans* models of mutations in these genes have been recently made, both by expressing mutant forms of the human proteins or by mutating the corresponding worm orthologues [7,9,14,24,25].

### 2.5. Other Neurological Disorders

Autism spectrum disorders are developmental diseases characterized by impaired social skills. In recent years, many candidate genes have been associated with these disorders, and in some cases, *C. elegans* models have been used to study them. That’s the case for the genes encoding neuroligin, neurexin, and shank, where mutants defective in the corresponding *C. elegans* orthologues of these genes, i.e., *nlg-1*, *nrx-1*, and *shn-1*, have been used to investigate their physiological role [11].

Amyotrophic lateral sclerosis belongs to a group of motor neuron diseases that also includes spinal muscular atrophy, primary lateral sclerosis, and hereditary spastic paraplegia, among others. *C. elegans* models with defects in the orthologues of several human genes which are known to be involved in these diseases are also available [24].

## 3. Dysregulation of Ca^2+^ Signaling in Aging and Neurodegeneration

The role of Ca^2+^ dysregulation in the development of neurodegenerative diseases, and more in general, in human and brain aging, has been investigated for nearly 40 years. The so called “Calcium Hypothesis of Alzheimer’s disease and brain aging” was first put forward in the 1980s, and has been reformulated several times since then on the basis on new data [26,27,28,29]. According to this hypothesis, when the mechanisms that normally control intracellular Ca^2+^ homeostasis in neurons undergo a sustained alteration that goes beyond the normal fluctuations in activity, this disruption of neuronal intracellular Ca^2+^ signaling may trigger brain dysfunction. This phenomenon would account for the age-associated decrease in cognitive functions, and also, in cases of larger alterations, could lead to the development of neurodegenerative diseases.

The evidence for this Ca^2+^ hypothesis has accumulated mainly in two ways. First, on the links between Aβ oligomers and tau and Ca^2+^ dysregulation, and second, on the effect of several mutations producing familiar Alzheimer’s disease on Ca^2+^ signaling. The Ca^2+^ hypothesis has been extended to cover many other neurodegenerative diseases, as well as normal aging. Several recent reviews have been published on this subject [27,28,29], and there are still many points under debate, but the main facts are as follows.

1. Many studies have shown that Aβ aggregates and tau induce significant increases in cytosolic [Ca^2+^]; several mechanisms have been reported for this effect, from the inhibition of Ca^2+^ extrusion through the plasma membrane Ca^2+^ ATPase or the Na/Ca^2+^ exchanger, to the activation of Ca^2+^ entry through voltage- or receptor-operated Ca^2+^ channels, and including the possible direct interaction of the Aβ oligomers with the lipid bilayer to form pores [29,30,31,32,33,34,35].

2. The effects of mutant presenilins on the Ca^2+^ content of the endoplasmic reticulum (ER) is still under debate. Early reports suggested that mutant presenilins increased the ER [Ca^2+^] level both by increasing the activity of the sarco-endoplasmic reticulum Ca^2+^ ATPase (SERCA) and by reducing the Ca^2+^ leak from the ER. In fact, it was reported that wild-type presenilins could, in part, mediate that leak by forming passive Ca^2+^-leak channels in the ER membrane. Mutant presenilins, instead, would have reduced Ca^2+^-leak activity [15,29,32,35,36]. However, recent data using fluorescent ER-targeted Ca^2+^ sensors have shown little change in the ER Ca^2+^ content in cells harboring Alzheimer’s disease-inducing PS1 mutations, or even a decreased ER Ca^2+^ content in the case of cells having Alzheimer’s disease-inducing PS2 mutations [37].

3. Aβ aggregates and presenilin mutations have also been shown to alter the function of the ER Ca^2+^ release channels, both ryanodine receptors (RyR), and inositol 1,4,5-trisphosphate receptors (IP_3_R), increasinh their sensitivity or expression level, and thus enhancing their Ca^2+^ release activity [27,29,38,39,40,41,42,43,44]. In contrast, the store-operated Ca^2+^ entry pathway is downregulated in Alzheimer’s disease models, although it is interesting to note that it has been shown to be upregulated in Huntington’s disease models [27,29,32,45,46].

4. Similar findings have been described in neuronal models of Parkinson’s disease, where α-synuclein aggregates trigger an increase in cytosolic [Ca^2+^] [47,48,49], which is preceded by a transient decrease due to activation of the SERCA pump by the aggregates [50]. This phase appears to be important for cell damage, as SERCA inhibitors were able to increase viability in cellular models and in *C. elegans* in vivo [48]. Studies in amyotrophic lateral sclerosis models also show an increased Ca^2+^ entry through AMPA-type glutamate receptors, increased ER Ca^2+^ release, and mitochondrial Ca^2+^ overload [51].

5. The mitochondria-associated ER membranes (MAMs) are specialized regions of the ER that are in close contact with mitochondria and seem to play a very important role in the pathogenesis of neurodegenerative diseases [52,53,54,55]. These regions constitute an almost direct pathway for Ca^2+^ transfer from the ER to mitochondria, thanks to the presence of IP_3_R in the ER, voltage-dependent anion channels in the outer mitochondrial membrane, and mitochondrial Ca^2+^ uniporters in the inner mitochondrial membrane, together with many other proteins that form long-lasting structures that keep ER and mitochondrial membranes at distances of 10–30 nm. It has been shown that presenilins and APP are ER integral membrane proteins that are enriched in the MAMs [56,57,58], so they can easily interact with all the machinery for ER-mitochondria Ca^2+^ transfer. In fact, several Alzheimer’s disease inducers or presenilin mutations enhance ER to mitochondria Ca^2+^ transfer through these structures [59]. MAMs are probably also important for other neurodegenerative diseases, such as Parkinson’s disease, Huntington’s disease, amyotrophic lateral sclerosis, Charcot-Marie-Tooth disease, Hereditary spastic paraplegias, or others, where several proteins associated with these diseases also accumulate in MAMs and are necessary for ER to mitochondria Ca^2+^ transfer [53,54,55,60]. Excess ER to mitochondria Ca^2+^ transfer may lead to mitochondrial Ca^2+^ overload and apoptosis, while a decreased Ca^2+^ transfer may impair mitochondrial metabolism. Disruption of mitochondrial Ca^2+^ homeostasis may thus also play a critical role in the development of neurodegenerative diseases [49,61].

6. Close contacts similar to the MAMs also appear to couple ER and lysosomes [62,63]. Disruption in Ca^2+^ homeostasis in lysosomes and on the ER-lysosomal contacts has also been proposed to be very important in the pathogenesis of several neurodegenerative diseases, in particular, amyotrophic lateral sclerosis, via either autophagy deregulation or changes in cellular Ca^2+^ dynamics [60,64]

## 4. Using the *C. elegans* Model to Study the Role of Ca^2+^ Signaling in Neurodegeneration

**Figure 1:**
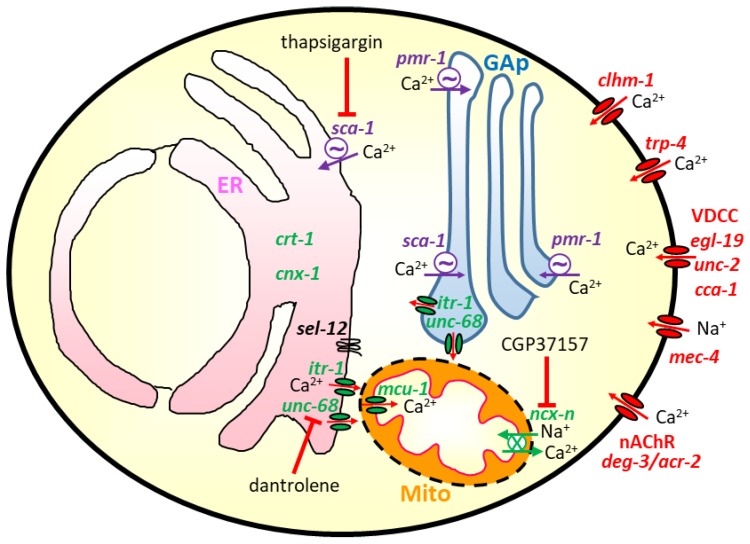
Scheme showing the Ca^2+^ pathways that have been shown to be involved in the modulation of aging and neurodegeneration in *C. elegans.* In red: genes that have been mutated to induce neurodegeneration by increasing Ca^2+^ entry from the extracellular medium; this includes Ca^2+^ entry through the *clhm-1* and *trp-4* Ca^2+^ channels, and through the nicotinic acetylcholine receptor nonselective cation channel with mutated *deg-3* or *acr-2* subunits. It also includes the mutated *mec-4* mechanosensitive Na^+^ channel, whose activation produces plasma membrane depolarization and activation of voltage-dependent Ca^2+^ channels such as *egl-19, unc-2,* or *cca-1*. In green: genes whose inactivation has been shown to produce protection in *C. elegans* models of neurodegeneration or increase longevity; this includes the Ca^2+^-binding ER chaperone proteins calreticulin (*crt-1*) and calnexin (*cnx-1*), the ER Ca^2+^ channels IP_3_R (*itr-1*) and RyR (*unc-68*), and the mitochondrial Ca^2+^ transporters, both the mitochondrial Ca^2+^ uniporter (*mcu-1*) and the mitochondrial Na^+^/Ca^2+^ exchanger (gene not yet identified within the large *C. elegans ncx-1 to 10* family [65]). Finally, in purple: genes whose modulation has produced in some cases neurotoxicity and in others neuroprotection; this group includes the SERCA Ca^2+^ pump (*sca-1*) and the Ca^2+^ pump of the secretory pathway (*pmr-1*, placed in the membrane of the Golgi apparatus (GAp)). The figure also shows some of the most used inhibitors of Ca^2+^ pathways, thapsigargin for the SERCA pump, dantrolene for the RyR and CGP37157 for the mitochondrial Na^+^/Ca^2+^ exchanger. The presenilin homologue *sel-12* gene is depicted in the ER membrane, close to the ER-mitochondria contacts.

As mentioned, the first *C. elegans* model for Alzheimer’s disease was created in 1995 [6], and since then, many other models for most neurodegenerative diseases have been created and are now available. These models have added much information on the role of Ca^2+^ signaling in aging and neurodegeneration. In this section, we will give a detailed account of the main findings obtained. The scheme in Figure 1 shows the main elements of the Ca^2+^ signaling toolkit in *C. elegans* worms that have been related to neurodegeneration and will be mentioned in this review.

### 4.1. The Role of ER Ca^2+^ Release in Neurodegeneration

Evidence for an important role of ER Ca^2+^ release in neurodegeneration was first obtained in *C. elegans* by studying the necrotic cell death induced by several gain-of-function mutations in ion channels [66,67,68]. The necrotic cell death caused by an hyperactive MEC-4 Na^+^ channel in *C. elegans* was abolished by loss-of-function mutations in either calreticulin (*crt-1*), the main ER Ca^2+^ binding chaperone protein, or in any of the Ca^2+^ release channels in the ER, the IP_3_R (*itr-1*) or the RyR (*unc-68*) [69]. However, *crt-1* mutation was unable to protect against the necrotic cell death induced by the increased Ca^2+^ influx induced by the mutant nicotinic acetylcholine receptor alpha subunit *deg-3*, which creates a highly permeable Ca^2+^ channel [69]. The conclusion from these initial studies was that a prolonged increase in cytosolic [Ca^2+^] is probably the final common step required for necrotic cell death. In some cases, Ca^2+^ influx to the cytosol is small and ER-Ca^2+^ release is required for necrosis (*mec-4* mutants), and in other cases, Ca^2+^ influx to the cytosol is enough to trigger necrosis (*deg-3* mutants) by itself. In this model, reducing cellular [Ca^2+^] with EGTA or inhibiting Ca^2+^ release through RyR with dantrolene reduced neurodegeneration, while SERCA inhibition with high concentrations of thapsigargin increased neurodegeneration by inhibiting Ca^2+^ reuptake into the ER [69].

A search for *C. elegans* mutants with defects in dopaminergic neurons identified strains with a progressive dopaminergic neuron loss during postembryonic life, which were then identified as having gain-of-function single amino acid mutations in the Transient Receptor Potential mechanosensory channel *trp-4* [70]. The phenotype was again clearly dependent on Ca^2+^ signaling dysfunction, as it was suppressed by loss-of-function mutations of the Ca^2+^ channels of the ER, either IP_3_R (*itr-1*) or RyR (*unc-68*), and by mutation of calreticulin (*crt-1*) [70]. Thus, the increased Ca^2+^ influx through mutant *trp-4* was probably increasing cytosolic and ER [Ca^2+^], leading to an increased ER Ca^2+^ release that resulted in the neuronal damage.

Similarly, a mutation in the *acr-2* subunit of the *C. elegans* nicotinic acetylcholine receptor led to loss of motor neurons and paralysis, which was reduced in the absence of both ER-Ca^2+^ binding chaperone proteins calreticulin (*crt-1*) and calnexin (*cnx-1*) (but not only one of them). However, even the protected double *cnx-1;crt-1* mutants in the presence of mutant *acr-2* finally underwent a delayed but progressive degeneration of motor neurons and paralysis [71]. Therefore, the intensity of the Ca^2+^ deregulation appears to be essential to determining whether modulation of ER-Ca^2+^ release can protect from neurodegeneration, and to what extent.

A polymorphism in the Ca^2+^ permeable ion channel CALHM1 has been associated with late-onset Alzheimer’s disease. The CALHM1 family has six members in humans, which complicates its study. *C. elegans* has only one homolog, *clhm-1*, which is expressed in the plasma membrane of body-wall muscle and some neurons, and has similar biophysical properties to human CALHM1 [72]. The overexpression of either human CALHM1 or *C. elegans clhm-1* in touch neurons induced neurodegeneration similar to that induced by the expression of the mutated *mec-4* channel. Neurodegeneration induced by *clhm-1* overexpression was delayed in calreticulin (*crt-1*) or calnexin (*cnx-1*) mutants, and in the presence of a Ca^2+^ chelator, indicating that it is mediated also by Ca^2+^ [72].

As mentioned in Section 2.4, mutations of the nuclear protein TDP-43 appear associated with some familial cases of amyotrophic lateral sclerosis. A *C. elegans* model expressing wild-type or mutant TDP-43 has been used to study the role of Ca^2+^ signaling in the paralysis and neurodegeneration of GABAergic motor neurons that appears in the mutants [73]. The results suggest an important role of ER-Ca^2+^ release in the development of the pathology. Paralysis and neurodegeneration in TDP-43^A315T^ mutant worms were suppressed by loss-of-function mutations of calreticulin (*crt-1*), calnexin (cnx-1), IP_3_R (*itr-1*), and RyR (*unc-68*). Dantrolene and incubation in the presence of the Ca^2+^ chelator EGTA also reduced neurodegeneration, while SERCA inhibition with high concentrations of thapsigargin increased it. It was suggested that the mutated protein TDP-43^A315T^ disrupts ER function, leading to an increase in cytosolic [Ca^2+^] that activates calpain and aspartyl proteases leading to cell necrosis [73].

A *C. elegans* model with overexpression of a tau mutation which is responsible for increased risk of frontotemporal dementia and Alzheimer’s disease was recently reported [74]. The transgenic worms showed a progressive neuronal loss in the glutamatergic system, which was significantly reduced by mutation of calreticulin (*crt-1*) or calnexin (*cnx-1*). This suggests that ER Ca^2+^ release is also a critical step in the mechanism of action of this tauopathy [74].

Reactive oxygen species (ROS) can also produce neurodegeneration. A model for ROS-dependent neurodegeneration has been established in *C. elegans* by expressing the genetically-encoded photosensitizer Killer Red in GABAergic neurons [75,76]. Illumination of the transgenic worms induces ROS production and triggers a neuronal cell death that morphologically resembles that induced by mutated ionic channels such as *mec-4*; furthermore, it is caspase independent and occurs through a necrosis-like process. Moreover, this ROS-mediated neurodegeneration was reduced in mutants of the IP_3_R (*itr-1*) and calreticulin (*crt-1*), although not in mutants of the RyR (*unc-68*) [75,76]. These results suggest that ROS-mediated neurodegeneration takes also place through a Ca^2+^-dependent mechanism.

*sel-12* is the *C. elegans* presenilin orthologue with the largest homology to human presenilins, and is also placed in the ER membrane [15]. The role of *sel-12* in Ca^2+^ signaling has been studied in detail. *sel-12* mutants had increased resting cytosolic [Ca^2+^] and increased cytosolic [Ca^2+^] activity that was dependent on ER-Ca^2+^ release. In addition, *sel-12* mutants showed mitochondrial morphological and functional defects which appeared to be dependent on the increased ER to mitochondria Ca^2+^ transfer, as they reverted either by knocking down the IP_3_R with RNAi, in RyR mutants (*unc-68*), or in the absence of the mitochondrial Ca^2+^ uniporter (*mcu-1*) [17].

The same group recently directly measured mitochondrial [Ca^2+^] using a targeted GCaMP6, and they showed a significant increase in the resting mitochondrial [Ca^2+^] in the *sel-12* mutants, both in body wall muscle cells and in several neurons; this was accompanied by an increased level of ATP and oxidative phosphorylation activity, and increased superoxide production. Again here, decreasing ER Ca^2+^ release or inhibiting mitochondrial Ca^2+^ uptake improved mitochondrial function and suppressed neurodegeneration in the *sel-12* mutants, suggesting that neurodegeneration in these mutants is caused by elevated ER to mitochondria Ca^2+^ transfer. [18]. Moreover, *sel-12* mutants show a proteostasis defect that leads to a premature protein aggregation in *C. elegans* models of polyglutamine, Aβ or mutant tau expression; this effect was suppressed by knocking down the IP_3_R with *itr-1* RNAi, in calreticulin (*crt-1*) mutants, or in *mcu-1* mutants [77]. These results suggest that the presenilin orthologue *sel-12* has a critical role regulating ER to mitochondria Ca^2+^ transfer, and *sel-12* mutations produce an increase of this Ca^2+^ transfer that plays a key role in the development of mitochondrial dysfunction, proteostasis, and neurodegeneration.

A *C. elegans* model of neurodegeneration was also generated by knocking down the pathway of coenzyme Q synthesis [78]. The subsequent mitochondrial dysfunction induced a selective degeneration of GABAergic neurons by a death mechanism that included characteristic features of both necrotic and apoptotic pathways. Importantly, this neuronal degeneration was suppressed by calreticulin (*crt-1*) knockdown or EGTA addition, indicating that mitochondrial dysfunction in this model triggers a pathway for neuronal degeneration that includes a Ca^2+^-dependent step, probably related with ER-Ca^2+^ release [78].

The role of Ca^2+^-dependent calpain proteases and lysosomal aspartyl proteases in the necrotic cell death that occurs during neuronal degeneration has received much support from data obtained in the *C. elegans* gain-of-function mutants of specific ion channels such as *mec-4* o *deg-3* [69,79,80]. According to the calpain-cathepsin hypothesis proposed by Yamashima [81,82], cytosolic Ca^2+^ overload induced by necrotic insults activates the Ca^2+^-dependent calpain proteases, which induce lysosomal membrane rupture and release of hydrolytic proteases, including cathepsins. In addition, the activation of calpains by deregulated Ca^2+^ homeostasis may also degrade nuclear pore components, increasing its permeability and altering nucleocytoplasmic trafficking. For example, in the *C. elegans* model of neuronal degeneration, due to the presence of mutated *deg-3*, it has been shown that calpain activation by cytosolic [Ca^2+^] induces the selective loss of a GFP protein fused to nucleoporin in the degenerating neurons [83]. Work in *C. elegans* has also provided evidence for the involvement of upregulated endocytosis and autophagy, together with proteolysis, to facilitate Ca^2+^-induced necrotic death and neurodegeneration [84,85].

### 4.2. The Role of SERCA in Aging and Neurodegeneration

In many of the *C. elegans* models of neurodegeneration mentioned above, inhibition of SERCA with thapsigargin potentiated neurodegeneration. However, in other models, SERCA inhibition showed a protective effect. As mentioned in Section 3, studies in neuronal models have shown that α-synuclein aggregates increase cytosolic [Ca^2+^] [47,48,49,50], but that this effect is preceded by a transient decrease due to activation of the SERCA pump by the aggregates [50]. In these models, a SERCA pump inhibitor had a neuroprotective effect. Similarly, in a *C. elegans* model of Parkinson’s disease overexpressing α-synuclein in dopaminergic neurons, a SERCA inhibitor rescued neuron survival to the level of the controls, without modifying the age-dependent neuronal loss in the controls [48]. These results suggest that SERCA activation may play an important role in the process of neuronal damage induced by α-synuclein, indicating that SERCA may be a possible therapeutic target for Parkinson’s disease.

Using a *C. elegans* model with Aβ overexpression in glutamatergic neurons, Griffin et al. [86] showed that the SERCA pump inhibitor thapsigargin reduced the neurodegeneration induced by Aβ overexpression, but had no effect in the controls. Further, similar findings were obtained using RNAi for *sca-1*, the *C. elegans* orthologue of SERCA. Interestingly, thapsigargin also induced neuroprotection in the presence of human APOEε4, but not in the presence of human APOEε2 or APOEε3, suggesting that ApoE has a role in Ca^2+^ homeostasis that is selectively lost by the APOEε4 allele, which is the allele that constitutes a very significant risk factor for Alzheimer’s disease in humans [86].

Following up on the effect of SERCA inhibitors, it has been recently shown that submaximal concentrations of SERCA inhibitors produced a significant increase in *C. elegans* lifespan [87]. Although the mechanism is still unclear, the increased lifespan could be attributed to a decreased ER [Ca^2+^], and therefore, reduced ER Ca^2+^ release after stimulation. Thus, reduced ER Ca^2+^ release could be a common mechanism promoting longevity and neuroprotection. In fact, it has been shown that SERCA (*sca-1* in *C. elegans*) is one of the proteins that undergoes a larger decrease in expression in *C. elegans* during aging, i.e., nearly 10-fold from day 4 to day 10 [88]. This decrease may be a physiological response to promote survival by reducing Ca^2+^ signaling. A higher SERCA content may be optimal in young individuals to allow a rapid response to external stimuli, but reducing SERCA activity at later ages may be important for survival.

### 4.3. The Role of the Secretory Pathway Ca^2+^ Pump ATPase

Work in *C. elegans* has led to the discovery of several important roles of the Golgi-localized Ca^2+^/Mn^2+^ pump *pmr-1* [89], the orthologous of the human secretory pathway Ca^2+^ ATPase (SPCA). Using a heat-stroke model in *C. elegans*, Kourtis et al. [90] found that preconditioning at 34 °C for 30 min increased the worm’s ability to survive after a subsequent heat-stroke at 39 °C. Importantly, preconditioning also protected against the necrotic cell death induced in *mec-4* and *deg-3* mutants, or after α-synuclein overexpression. This effect was mediated by Ca^2+^ because both intrinsic and acquired resistance to the heat-stroke was increased by reducing Ca^2+^ release through either IP_3_R (with RNAi against *itr-1*) or RyR (by inhibition with dantrolene). The PMR-1 pump was involved in this phenomenon, because *pmr-1* deficiency suppressed preconditioning-acquired resistance to heat stroke, while the overexpression of *pmr-1* protected against heat-stroke, even without preconditioning. Actually, a sudden heat stroke sharply increased cytosolic [Ca^2+^] in *C. elegans* neurons, and this effect was blocked by 34 °C preconditioning, by reducing Ca^2+^ release through either IP_3_R (*itr-1* RNAi) or RyR (dantrolene), or by inhibiting SERCA (*sca-1* RNAi). Instead, it was enhanced in *pmr-1* null mutants. Thus, Ca^2+^ pumping into the Golgi by the PMR-1 Ca^2+^ pump is very important for survival after heat stroke, an effect which was conserved also in mammalian neurons. Importantly, PMR-1-dependent preconditioning was also efficient at protecting against several neurotoxic stimuli, including α-synuclein overexpression [90].

Contrarily to the protective effect of the PMR-1 pump against heat-stroke, expression of α-synuclein in dopaminergic neurons of *C. elegans* produced neuronal death and an increase of the resting cytosolic [Ca^2+^]; both effects were prevented in *pmr-1* deficient mutant nematodes [91]. This effect of PMR-1 enhancing the α-synuclein toxicity is phylogenetically conserved, as it was also observed in yeasts and flies [91]. Therefore, PMR-1-dependent preconditioning protected against α-synuclein toxicity, but the presence of PMR-1 is necessary for α-synuclein toxicity. A possible explanation that has been proposed for these contrasting effects of PMR-1 activity could be that a transient activation of PMR-1, as in via preconditioning, may be cytoprotective, while a persistent deficit of PMR-1 may protect from α-synuclein toxicity by a different mechanism. It is unclear, for example, if PMR-1 could be involved in Ca^2+^-dependent α-synuclein secretion to the extracellular medium [89]. In any case, the precise mechanism of each effect is still unknown.

PMR1 also has an important role in embryogenesis [92], and *pmr-1* mutants show important defects in cell migration and attachment that resemble the loss of skin cell adhesion observed in Hailey-Hailey disease, a dominant human disease caused by the loss of one of the copies of the SPCA1 gene [93]. Interestingly, the level of embryonic lethality could be modulated by changing the activity of IP_3_R and RyR channels, although the modulation of each of these channels produced opposite effects. Double mutants of *pmr-1* and a gain of function mutant of *itr-1* (the worm IP_3_R orthologue) reduced lethality, and double mutants of *pmr-1* and a loss-of-function mutant of *itr-1* showed enhanced lethality or were not viable. In contrast, depletion with RNAi of *unc-68* (the worm orthologue of RyR) considerably increased the viability of *pmr-1* mutants [92]. Although difficult to explain, these findings strongly suggest that Ca^2+^ signaling play a critical role in cell migration and attachment during embryogenesis.

### 4.4. Models of Neurodegeneration Induced by Neurotoxins

One of the most used models of Parkinson’s disease in *C. elegans* is the dopaminergic neurons death induced by neurotoxins such as 6-hydroxydopamine (6-OHDA) or 1-methyl-4-phenyl- 1,2,3,6-tetrahydropyridine (MPTP). Both compounds are selectively accumulated by dopaminergic neurons through the dopamine transporter *dat-1*, expressed only in dopaminergic neurons, and they produce damage in few hours [94]. The *dat-1* transporter is essential for toxicity, and dopaminergic neurons in *dat-1* mutants are insensitive to these neurotoxins. However, these toxins are not specific for dopaminergic neurons, and also produce damage in other cell types that leads to decreased survival, even in *dat-1* mutants [95]. The vertebrate divalent metal transporter (DMT-1) orthologues *smf-1* and *smf-2* also contribute to 6-OHDA toxicity, and *smf-1* and *smf-2* mutants are more resistant to 6-OHDA toxicity [96]. As 6-OHDA damages neurons by inducing oxidative stress, it has been proposed that these transporters probably act by carrying out Fe^2+^ or Mn^2+^ uptake, thus providing a catalyst for the Fenton or Haber-Weiss reactions to produce further ROS and neuronal damage [96]. Interestingly, and in contrast to models of Ca^2+^-induced necrotic cell death [69,70], 6-OHDA-induced neurodegeneration was increased in *C. elegans crt-1* (calreticulin) mutants, and it was not modified in *itr-1* (IP_3_R) mutants [97].

Manganese is an essential oligoelement that works as a cofactor of several enzymes such as transferases, hydrolases, and superoxide dismutases. However, excess intake of Mn^2+^ is toxic and produces manganism, a progressive condition with symptoms very similar to Parkinson’s disease. In *C. elegans*, Mn^2+^ toxicity induces mitochondrial dysfunction, ROS production, mitochondrial and ER unfolded protein response, and increased protein misfolding [98]. Cellular Mn^2+^ transport to the cytosol requires *smf-1*, one the *C. elegans* orthologues of DMT-1, which is expressed in dopaminergic neurons. Consequently, *smf-1* mutants are protected against neuronal death induced by Mn^2+^ [96].

Then, once Mn^2+^ has entered the cytosol in dopaminergic neurons, it uses known Ca^2+^ pathways to get access to organelles and produce toxicity. Mn^2+^-induced mitochondrial dysfunction is due to the entry and accumulation of Mn^2+^ in the mitochondrial matrix through the mitochondrial Ca^2+^ uniporter, because *C. elegans* worms lacking MCU (*mcu-1* mutants) are more resistant to Mn^2+^ toxicity [99]. In addition, Mn^2+^ toxicity may also be related to its transport and accumulation by the PMR-1 Golgi resident Ca^2+^ pump, which has also a high affinity for Mn^2+^ and transports both cations with the same efficiency [89].

The widely-used metal Al^3+^ also induces dopaminergic neuron degeneration in *C. elegans*, and Al^3+^ exposure has been related in humans with the development of Parkinson’s or Alzheimer’s disease. *C. elegans* has three orthologues to DMT-1, named *smf1-3*, all of which are expressed in dopaminergic neurons. While *smf-1* is responsible for Mn^2+^ transport, *smf-3* is responsible for Al^3+^ transport to the cytosol, and *smf-3* mutants are resistant to Al^3+^ toxicity [100].

### 4.5. Role of Glial Cells in Neurodegeneration

A *C. elegans* hermaphrodite contains 302 neurons and 56 glial cells. Each glial cell keeps an invariant association with specific neurons, making this nematode a very exciting model with which to study glia-neuron interactions. In addition, recent work has shown that many aspects of glia-neuron interactions keep strong similarities in nematodes and higher animals [101].

The role of glial cells in neurodegeneration is increasingly recognized, and work on *C. elegans* is set to contribute significantly to the understanding of the mechanism of this interaction. A very interesting model for glial control of Ca^2+^-dependent neuronal degeneration has been recently described in *C. elegans*. Mutation of the *swip-10* gene (with still unknown function) induced hyperexcitability and a progressive degeneration of dopaminergic neurons, which was suppressed only by glial (but not neuronal) expression of the wild-type *swip-10* gene [102,103]. Dopaminergic neuron degeneration in *swip-10* mutants was reduced in calreticulin (*crt-1*) mutants and in calpain (*clp-1*) mutants, suggesting that excessive Ca^2+^ increases are involved in the process. In addition, neurodegeneration was also attenuated by mutations in glutamate vesicular and plasma membrane transporters (*vglu-3 and aat-1*), and by mutations in the Ca^2+^-permeable glutamate receptors *nmr-2* (NMDA-type) and *glr-1* (AMPA-type) present in dopaminergic neurons [103]. The data suggest a picture in which *swip-10* mutation somehow perturbs glutamate transport in glial cells, leading to an increased activation of neuronal glutamate receptors, increased neuronal Ca^2+^ signaling, and progressive neurodegeneration by apoptosis.

It has also been shown in *C. elegans* worms that some neuropeptides released by glial cells act on neuronal receptors, and that they somehow modulate age-related decline in mating, feeding, and locomotion. In fact, natural variation by single-gene polymorphisms in these neuropeptides explains half of the variation in age-related decline in these parameters among different strains [104]. This opens the way in the search for similar neuropeptides in humans, as they may be able to modulate healthy lifespan.

## 5. *C. elegans* Models to Search for Treatments to Neurodegenerative Diseases

*C. elegans* models of neurodegenerative diseases have been used to search for small molecule possible treatments. The amyotrophic lateral sclerosis model with mutant TDP-43 ^A315T^ has been used recently to do a phenotypical screening for potential therapeutic compounds [105]. After screening 3850 small molecules, 13 compounds showed positive effects, all of which were neuroleptics, with the most potent being pimozide. This positive effect was later confirmed in zebrafish and mice models, and in a clinical trial of sporadic amyotrophic lateral sclerosis patients. Regarding the mechanism of action, it was found that pimozide mainly acted through the inhibition of T-type Ca^2+^ channels in both *C. elegans* and zebrafish models [105]. Interestingly, L-type Ca^2+^ channel agonists FPL 64176 or Bay K 8644 had been shown before to protect the same zebrafish amyotrophic lateral sclerosis model against neuromuscular dysfunction [106]. In contrast, a screening of 38 neuroprotective compounds was recently made in a *C. elegans* model of amyotrophic lateral sclerosis, with null mutation of *dnc-1*, the homologous of human dynactin-1, and the L-type Ca^2+^ channel blocker nifedipine greatly improved motor deficits, as well as axonal degeneration [107]. Although the mechanism is probably complex, these results clearly indicate that Ca^2+^ signaling plays a very important role in the pathogenic mechanism of this disease.

Batten or CLN3 disease is a pediatric progressive neurodegenerative disease whose symptoms start at 4–7 years of age. It is due to recessive mutations in the CLN3 gene, and the pathogenesis is unclear. The CLN3 protein is a desaturase of palmitoylated membrane proteins, but the substrates are not well known. However, there is evidence of an intracellular Ca^2+^ dysregulation leading to apoptosis. Three homologues of CLN3 exist in *C. elegans* (*cln3-1*, *cln3-2* and *cln3-3*). A *cln-3* triple knockout *C. elegans* model for this disease has been made, and it shows a significant decrease in lifespan and increased mitochondrial mass. This model was used to make a screening for the effect of several Ca^2+^ channel inhibitors; it was found that the L-type Ca^2+^ channel inhibitor flunarizine rescued the short lifespan and prevented mitochondrial mass increase [108].

CGP37157, an inhibitor of the mitochondrial Na^+^/Ca^2+^ exchanger that has shown neuroprotective activity in several experimental models of neurotoxicity [109,110], was recently reported to be able to increase *C. elegans* lifespan by a mechanism that required functional mitochondria, because the effect disappeared in the *nuo-6* mitochondrial complex I mutant strain [111].

High-throughput drug screening technologies using *C. elegans* to search for compounds that modify aging or neurodegeneration are increasingly being used [7,8,105,107]. Some of them have been made in liquid media or in microwell plates, but more recently, microfluidic devices have emerged as powerful tools for these large-scale screenings [112]. In some cases, Ca^2+^ signaling has been used as a reporter for screening. For example, one of these high-throughput microfluidic assays was shown to be able to make an automatic quantification of the calcium transients of the ASH neurons at a rate of 400–450 worms/h, and it was used to screen a library of FDA-approved compounds to identify compounds able to prevent age-dependent neuronal functional decline [113]. These methods have a great potential to boost drug discovery in the future, using the variety of *C. elegans* disease models which are now available. The present evidence suggests that Ca^2+^ flux pathways are promising targets in the search for therapies to treat neurodegenerative diseases or to delay aging.

## 6. Direct [Ca^2+^] Measuring in *C. elegans* in Vivo

Very precise methods to directly monitor [Ca^2+^] in defined cells of worms are now available, and should be increasingly used in the coming years to obtain further and more precise information. Optical imaging of Ca^2+^ dynamics in *C. elegans* is possible during the whole life of the animal because it is always transparent. The first dynamic cytosolic [Ca^2+^] measurements were obtained in 2000 using a genetically-encoded Ca^2+^ indicator expressed in pharyngeal muscle [114]. Since then, a variety of fluorescent Ca^2+^ sensors have been used to monitor [Ca^2+^] in pharynx, vulva, and body wall muscles, as well as in several kind of neurons [115,116], and also in subcellular compartments such as mitochondria [117]. These studies have made it possible to monitor the muscle or neuronal dynamics in live worms, either in resting conditions or after pharmacological or optogenetic stimulation.

The changes in the dynamics of cytosolic and mitochondrial Ca^2+^ signaling at different times from day 2 to day 12 of worm age were recently measured in pharynx muscle [117,118]. Long-lasting Ca^2+^ records in live, nonanesthetized worms showed a fast spiking activity that persisted during aging, in spite of the known decline in pharynx pumping with age. This suggests that the [Ca^2+^] signal in the pharynx muscle cells of old animals is better maintained than muscle contraction, probably because of sarcopenia. Young animals, however, showed with higher frequency long periods of persistent high [Ca^2+^] during serotonin stimulation, either by excess stimulation of a very active Ca^2+^ entry/release machinery, or by excess energy waste through Ca^2+^ pumping and contraction leading to energy depletion [118]. Importantly, mitochondria were able to follow the high frequency oscillations of cytosolic [Ca^2+^] at all ages, showing that the fast mechanisms of uptake and the release of Ca^2+^ from mitochondria remain intact during aging [117].

In recent years, more sophisticated approaches have made it possible to perform whole-brain Ca^2+^ imaging experiments, where about 100 neurons are simultaneously monitored in a moving worm [119,120]. The application of these powerful techniques to the available models of neurodegenerative diseases in *C. elegans* will surely shed new light on the mechanisms and possible treatments of neurodegeneration.

## 7. Conclusions

The ease of genetic management of the nematode *C. elegans* has made it possible to generate models of essentially any neurodegenerative disease. The existing models recapitulate many of the characteristics of the disease at the cellular level, and the ability we have to easily alter the expression of any gene provides enormous possibilities for the study of the disease mechanisms. In addition, it is possible in this model to do high throughput screening of large series of drugs, and in many cases, the compounds that are effective in this model are also equally effective in other, higher-order animal models.

Regarding the research for the role of Ca^2+^ signaling in neurodegeneration, experiments in *C. elegans* worms have provided much information, either thanks to the ability to alter the expression of genes related to calcium fluxes (e.g., calreticulin, *crt-1*; calnexin, *cnx-1*; IP_3_R, *itr-1*; RyR, *unc-68*; SERCA, *sca-1*; SPCA, *pmr-1*), or to the possibility of adding compounds to the culture medium that modify these pathways. At the same time, the model offers the possibility of direct monitoring of [Ca^2+^] in defined cell populations of live animals, or even in subcellular organelles. The scheme in Figure 1 shows the intracellular location of the main Ca^2+^ signaling mechanisms related to neurodegeneration mentioned in this review.

The evidence available indicates that many Ca^2+^ pathways are involved in the process of neurodegeneration, including Ca^2+^ uptake into the ER (SERCA, *sca-1*), or into the Golgi apparatus (SPCA, *pmr-1*), Ca^2+^ release from ER, or Golgi through IP_3_R (*itr-1*) or RyR (*unc-68*), Ca^2+^ uptake into mitochondria through the MCU (*mcu-1*) and Ca^2+^ entry through several channels of the plasma membrane (nicotinic acetylcholine receptor, *trp-4, clhm-1*). The effects of modulation of these pathways in neurodegeneration are complex and sometimes contradictory in the different models. The activation of plasma membrane Ca^2+^ channels always induces neurodegeneration. Then, depending of the intensity of the activation of Ca^2+^ entry, the knock-down of genes for ER Ca^2+^ storage (*crt-1, cnx-1*), ER Ca^2+^ release channels (*itr-1* or *unc-68*) or mitochondrial Ca^2+^ uptake (*mcu-1*) may be protective or not. Similarly, the inhibition of the *sca-1* or *pmr-1* Ca^2+^ ATPase pumps may be protective or increase neurodegeneration, depending of the model. Therefore, it is clear that intracellular Ca^2+^ signaling plays a critical role in the process of neurodegeneration. However, the system is quite complex, and there is only a thin line separating the level of calcium fluxes that can be considered normal and those that may lead to pathology.

An example of this thin line can be found in the changes in mitochondrial [Ca^2+^] occurring during ER to mitochondria Ca^2+^ transfer in the mitochondria associated ER membranes (MAMs). Many models of neurodegeneration include mitochondrial damage as one of their characteristic features. An increase in ER Ca^2+^ content/release always produces an increase in mitochondrial [Ca^2+^] via Ca^2+^ transfer through the MAM structures. A certain degree of ER to mitochondria Ca^2+^ transfer may serve to increase mitochondrial metabolism and ATP production, and this may be protective. However, excess Ca^2+^ transfer may lead to mitochondrial Ca^2+^ overload and damage.
